# Deep drilling on Mars: pneumatic chip clearing model for the RedWater mining system

**DOI:** 10.1007/s44461-026-00013-y

**Published:** 2026-08-28

**Authors:** Leo Stolov, Joseph Palmowski, Kris Zacny, Bernice Yen, Bolek Mellerowicz, Zach Mank, Luke Sanasarian, Jack Schultz

**Affiliations:** 1 Honeybee Robotics, 2408 Lincoln Ave, Altadena, CA 91001 USA; 2 Center for Space Resources, Colorado School of Mines, Golden, CO United States

**Keywords:** In-situ resource utilization (ISRU), Pneumatic cuttings transport, Coiled tube drilling, Planetary drilling, Dilute-phase pneumatic conveying, Mars, Thermal vacuum testing, Water ice mining, Choking velocity

## Abstract

Mining water ice on Mars is a vital, near-term step to enable in-situ resource utilization (ISRU) and sustainable human exploration. Large quantities of water are required for propellant production and life support systems. Recent analysis of orbital data points to vast buried ice deposits in the Martian mid-latitudes. The RedWater system is a deep drilling and water-well system engineered to access and extract water from these ice deposits. RedWater accesses the buried ice by drilling through the overburden and using compressed gas to pneumatically clear the drill cuttings, or chips, out of the resulting borehole. Unlike terrestrial drilling, where heavy water-based slurries remove cuttings, Martian operations demand low-mass, gas-driven solutions robust to the planet’s low temperatures and atmospheric pressures. This paper presents a pneumatic chip clearing model for deep drilling operations on Mars, quantifying the minimum gas mass flow rates necessary to efficiently transport drill cuttings. The model applies terrestrial, pneumatic vertical conveying correlations and tests them against a range of Mars-relevant conditions. Experimental validation includes a dedicated test setup in vacuum, and two tests with the end-to-end RedWater system. The model shows reasonable agreement with experimental results in predicting gas flow requirements, providing useful guidance for mission architectures utilizing pneumatic drilling and mining on Mars

## Introduction

Mining water ice on Mars is critical for future in-situ resource utilization and eventual human missions. A propellant plant on Mars could demand 150 t of water to fuel a Mars ascent vehicle (Oleson et al. 2024). Refueling Starship requires approximately 600 t of water (Heldmann et al. 2022). Mining this ice requires finding and accessing large ice deposits.

**Table:** Nomenclature used for pneumatic chip clearing model

Group	Parameter	Nomenclature	Units
RedWater properties	Borehole diameter	$\:{D}_{borehole}$	m
	RedWater diameter	$\:{D}_{RW}$	m
	Borehole depth	$\:\:L$	m
	Hydraulic diameter	$\:{D}_{H}$	m
	Hydraulic area	$\:{A}_{H}$	m^2^
	Annulus area	$\:{A}_{A}$	m^2^
	Rate of Penetration	* ROP*	m/s
Gas properties	Gas density	$\:\rho\:$	kg⋅m^− 3^
	Dynamic viscosity	$\:\eta\:$	Pa⋅s
	Gas temperature	* T*	K
	Pressure at the top of the hole, or ambient pressure	$\:{P}_{tophole}$	Pa
	Pressure drop due to frictional losses	$\:{P}_{friction}$	Pa
	Hydrostatic pressure in the borehole	$\:{P}_{hydrostatic}$	Pa
	Pressure at the bottom of the borehole	$\:{P}_{borehole}$	Pa
Drill chip properties	Chip density	$\:{\rho\:}_{p}$	kg/m^3^
	Sphericity	$\:\psi\:$	–
	Chip Equivalent Diameter	* d*	m
	Mass flux	* j*	kg⋅s^− 1^⋅m^− 2^
Environmental properties	Gravity	* g*	m/s^2^
Dirty flow properties	Chip free fall velocity	$\:{w}_{fo}$	m/s
	Sphericity-corrected chip free fall velocity	$\:{w}_{fo\psi\:}$	m/s
	Chip Reynolds Number	* Re*	–
	Archimedes number	* Ar*	–
	Froude number	* Fr*	–
	Dimensionless particle diameter	$\:{d}^{*}$	–
	Dimensionless gas velocity	$\:{u}^{*}$	–
	Choking gas velocity	$\:{v}_{c}$	m/s
	Voidage	*ε*	–
	Choking voidage	$\epsilon_{c}$	–
	Friction factor	$\:\lambda\:$	–
	Factor of Safety	* FOS*	–
	Design velocity	$\:{v}_{design}$	m/s
Input gas flow properties	Upstream gas density	$\:{\rho\:}_{0}$	kg/m^3^
	Upstream gas pressure	$\:{P}_{0}$	Pa
	Orifice discharge coefficient	$\:{C}_{D}$	–
	Orifice open area	$\:{A}_{0}$	m^2^
	Heat capacity ratio	$\:\gamma\:$	–
	Gas mass flow rate	$\:\dot{m}$	kg/s

### Mars buried ice

**Fig. 1:**
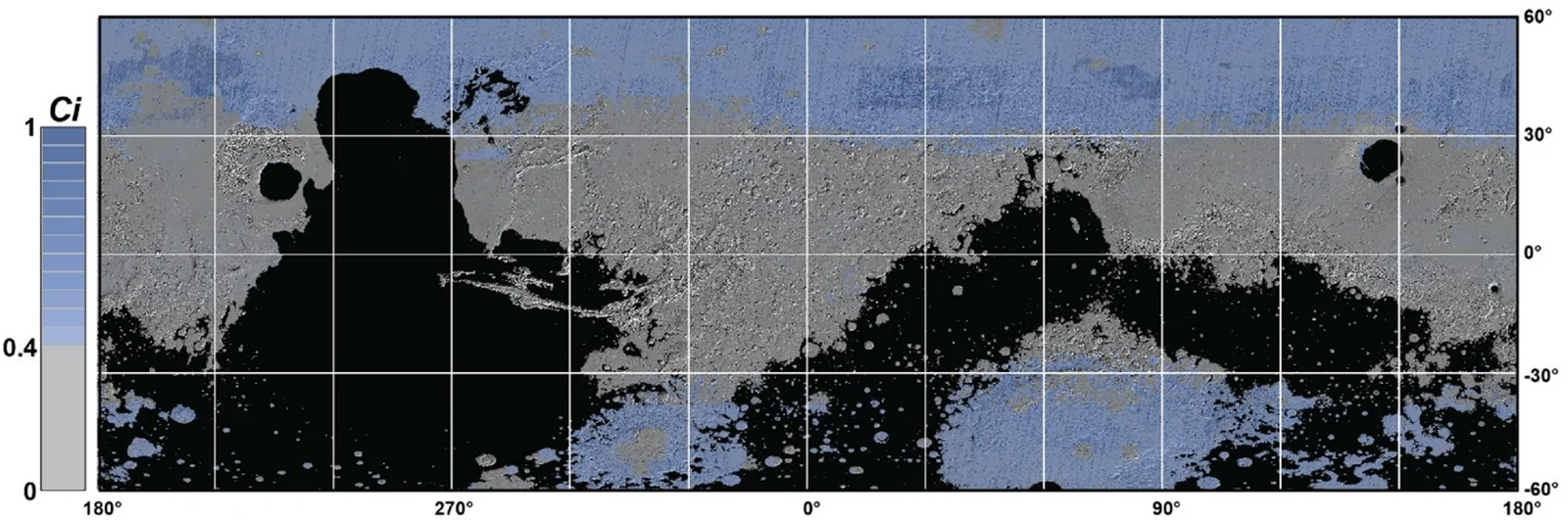
Ice consistency map of Mars from the SWIM study for depths of 1–5 m. Shades of blue show the ice consistency index (Ci), the degree to which five ice-characterization techniques are consistent with the presence of ice (0 indicates no data or ambiguity; 1 indicates full consistency across all techniques). Black areas represent elevations above 1 km. Note. Adapted from Morgan et al. (2025), The Planetary Science Journal, 6(5), Article 107. https://doi.org/10.3847/PSJ/ad9b24. CC BY 4.0.

Orbital data of the Martian mid-latitudes reveal extensive deposits of shallow ground ice buried under centimeters to meters of regolith overburden. The Subsurface Water Ice Mapping project (SWIM) used data from CTX, SHARAD, TES, and other instruments to create a distribution map of buried ice such as the one shown in Fig. 1 (Morgan et al. 2025). In certain regions, erosion at several mid-latitude scarps exposes thick, massive ice deposits with very low dust content, beginning only 1–2 m below the surface and extending downward for more than 100 m (Dundas et al. 2018).

### RedWater system overview

**Fig. 2:**
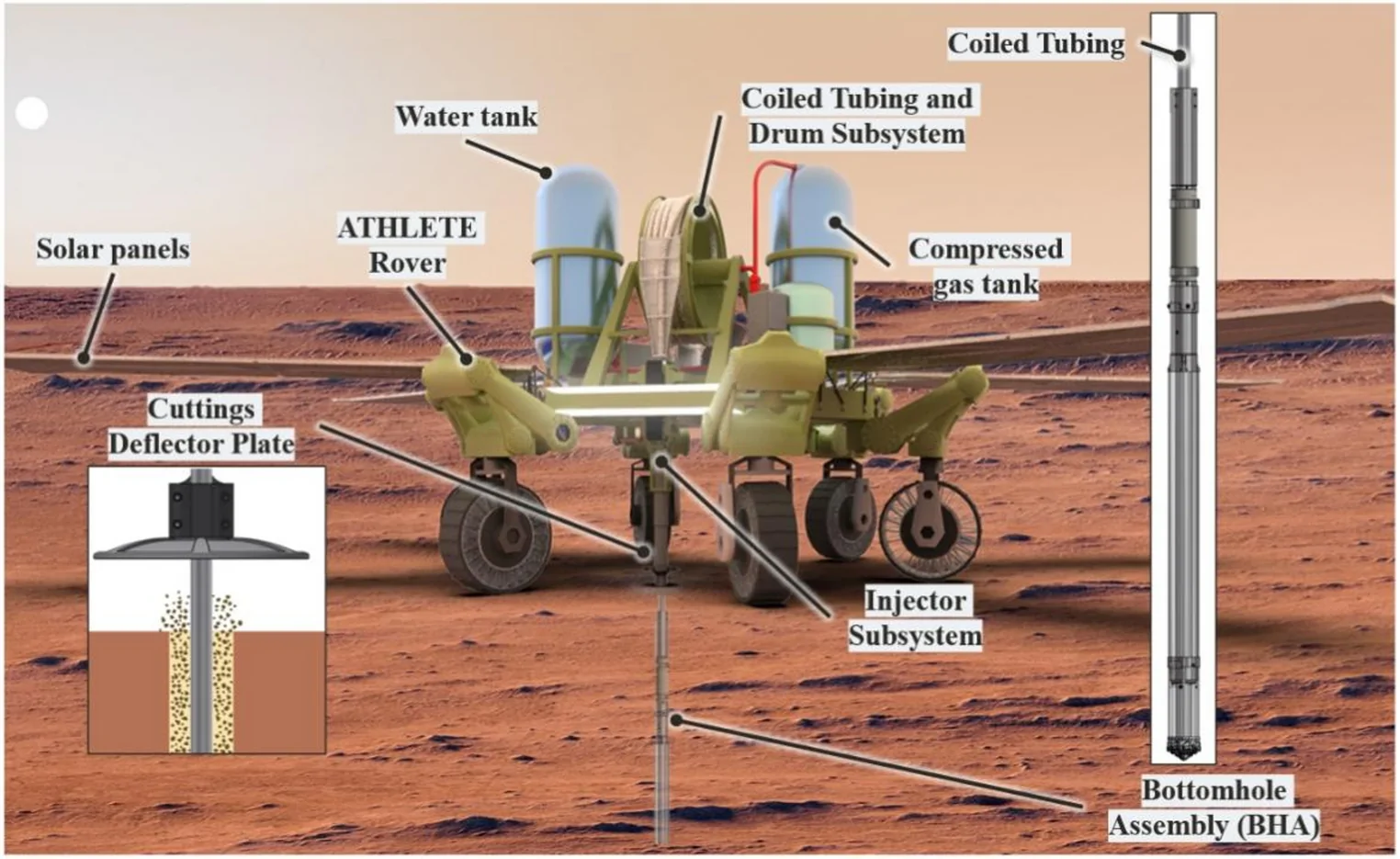
Overview of RedWater subsystems. RedWater is shown mounted on the ATHLETE system to showcase a potential mission concept

RedWater, developed and tested at Honeybee Robotics, is a system designed to mine water at up to 25 m in depth on Mars. RedWater works by drilling through the rocky overburden, melting the buried ice, and pumping it up to the surface. Major subsystems are shown in Fig. 2.

In order to mine large quantities of water, RedWater uses Rodriguez Well (RodWell) technology (Rodriguez 1963). The Rodwell is a type of well traditionally used to extract water in polar areas on Earth. It works by melting a subsurface cavity in an ice deposit and continuously recirculating in heated water to quickly grow the well. Water is intermittently siphoned off once the well is large enough. At Amundsen-Scott South Pole Station in Antarctica, Rodwell technology has been providing water since 1995 up to present day (National Science Foundation, Office of Polar Programs 2025). The success of this approach is a big reason why RodWell technology has been proposed and studied intensively for mining water on Mars (Hoffman et al. 2020).

**Fig. 3:**
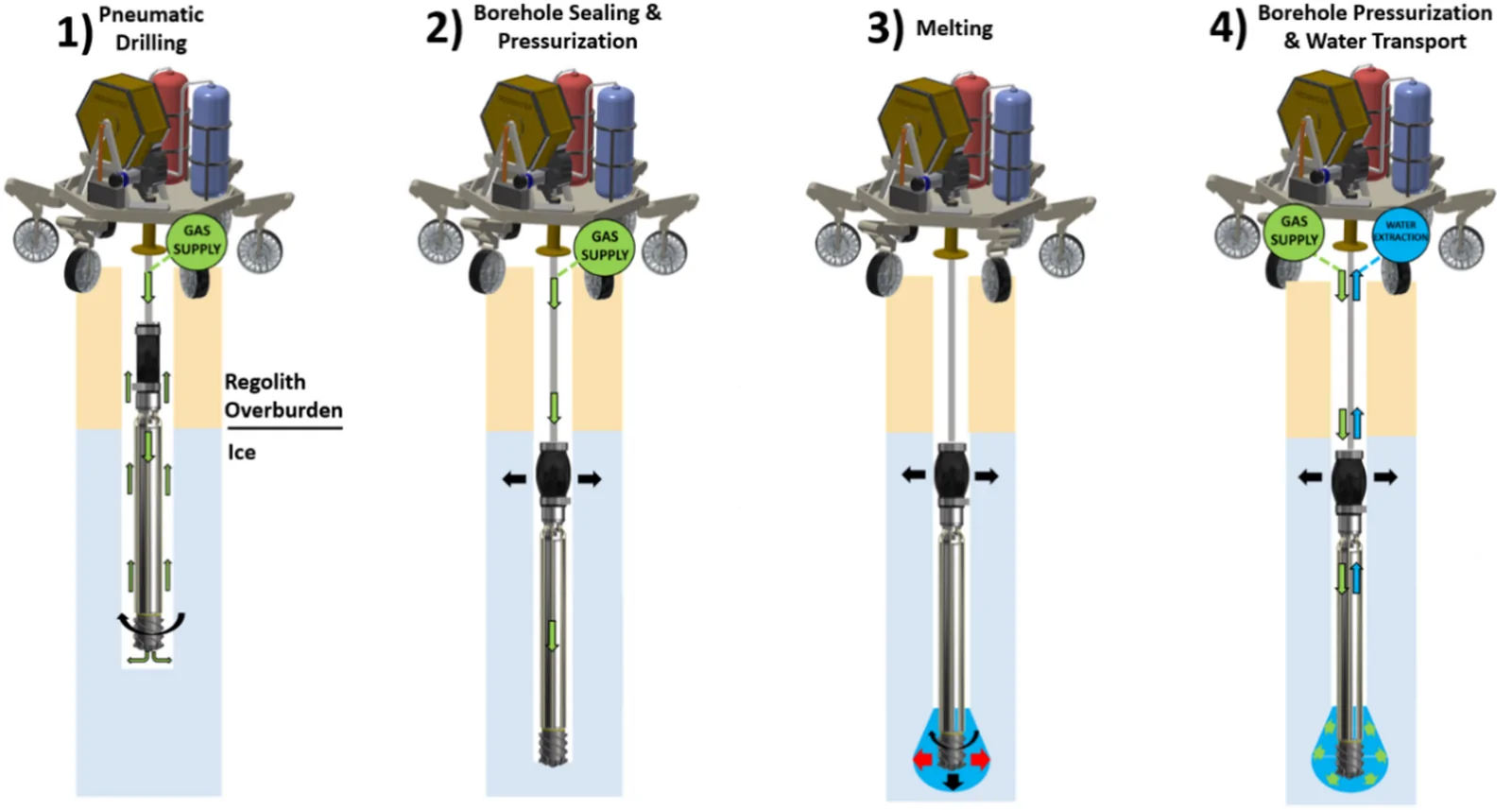
Overview of the RedWater concept of operations. (1) Pneumatic drilling: the injector system pushes the coiled tubing and BHA through the rock and ice. Rotary percussive drilling breaks apart the material as it is picked up and blown out of the borehole pneumatically. (2) An inflatable packer seals the borehole and gas pressurizes the annulus. (3) Heaters inside the BHA melt the ice. (4) Water is pumped up from the subsurface pool and heated in a top-hole heat exchanger, then recirculated back down into the hole to melt more ice. Water pumping continues, with some fraction of water harvested with each recirculation

None of this is possible without first accessing the ice. To drill through the overburden, RedWater uses a rotary percussive drill as part of the Bottom Hole Assembly (BHA) to break the rock and ice into small chips. Compressed gas is used to pick up and transport these chips up the borehole wall to the surface. To deploy and preload the BHA up to a 25 m depth, RedWater uses coiled tubing technology, which is commonly used for deep drilling on Earth. Coiled tubing uses a metallic hollow tube that is plastically deformed around a drum and deployed with the use of an injector. The tube reacts drilling loads and acts as a conduit for electrical, pneumatic, and hydraulic lines to run from the surface down to the BHA. The drilling and water extraction steps are shown in Fig. 3. More details on this system and its performance are described in Palmowski et al. (2023, 2024) and Mellerowicz et al. 2022.

While RedWater adapts terrestrial drilling heritage, it has been designed for the Martian environment and miniaturized for integration with rover-class mobility platforms such as JPL’s All-Terrain Hex-Limbed Extra-Terrestrial Explorer (ATHLETE) (Wilcox 2012). RedWater reduces sensitivity to environmental unknowns such as rocky overburden composition and ice-deposit depth by mechanically penetrating the overburden before melting the ice. This is a key distinction from conventional ice-penetration concepts for icy worlds, such as melt probes, which are generally optimized for ice-rich deposits and can be obstructed by non-ice heterogeneities expected in the Martian mid-latitudes including dust layers, sediments, and rocks (Dachwald et al. 2023). RedWater uses a mechanical rotary-percussive/pneumatic drilling phase before initiating subsurface melting and water extraction from the ice deposit (Fig. 3).

### RedWater pneumatic drilling

This paper focuses on the pneumatic drilling operations of the RedWater system (step 1 in Fig. 3). The rotary percussive system in the BHA is designed to break up rock and ice into small cuttings or drill chips. As drill chips are created, they must be cleared out of the borehole; if they build up, drilling energy increases until the drill eventually stalls (Bar-Cohen and Zacny 2020). On Earth, liquid drilling muds are the most common method for transporting chips to the surface, though air and gas systems are also used in hard rock applications (Lyons et al. 2020). However, these slurries are typically heavy and water-based – poor properties for the Martian environment as systems need to be low mass and robust to extreme temperatures. Instead, RedWater uses compressed gas as the drilling fluid. Compressed gas is an effective chip-clearing method in the low-pressure Martian atmosphere (~ 600 Pa), where a given supply pressure produces a larger pressure ratio than it would on Earth. This larger pressure ratio can generate higher gas velocities near the bit, increasing drag and momentum transfer to the drill chips and helping entrain them and carry them out of the borehole.

Pneumatic drilling can be conducted using either reverse circulation or direct circulation methods (Lyons et al. 2020). In reverse circulation, cuttings are transported up through the center of the drill pipe. This is a particularly effective method in unstable formations (Cui et al. 2025) but comes at the cost of complexity for a drill design. In direct circulation drilling, compressed gas flows down the drill string and returns upward through the annulus carrying cuttings.

**Fig. 4:**
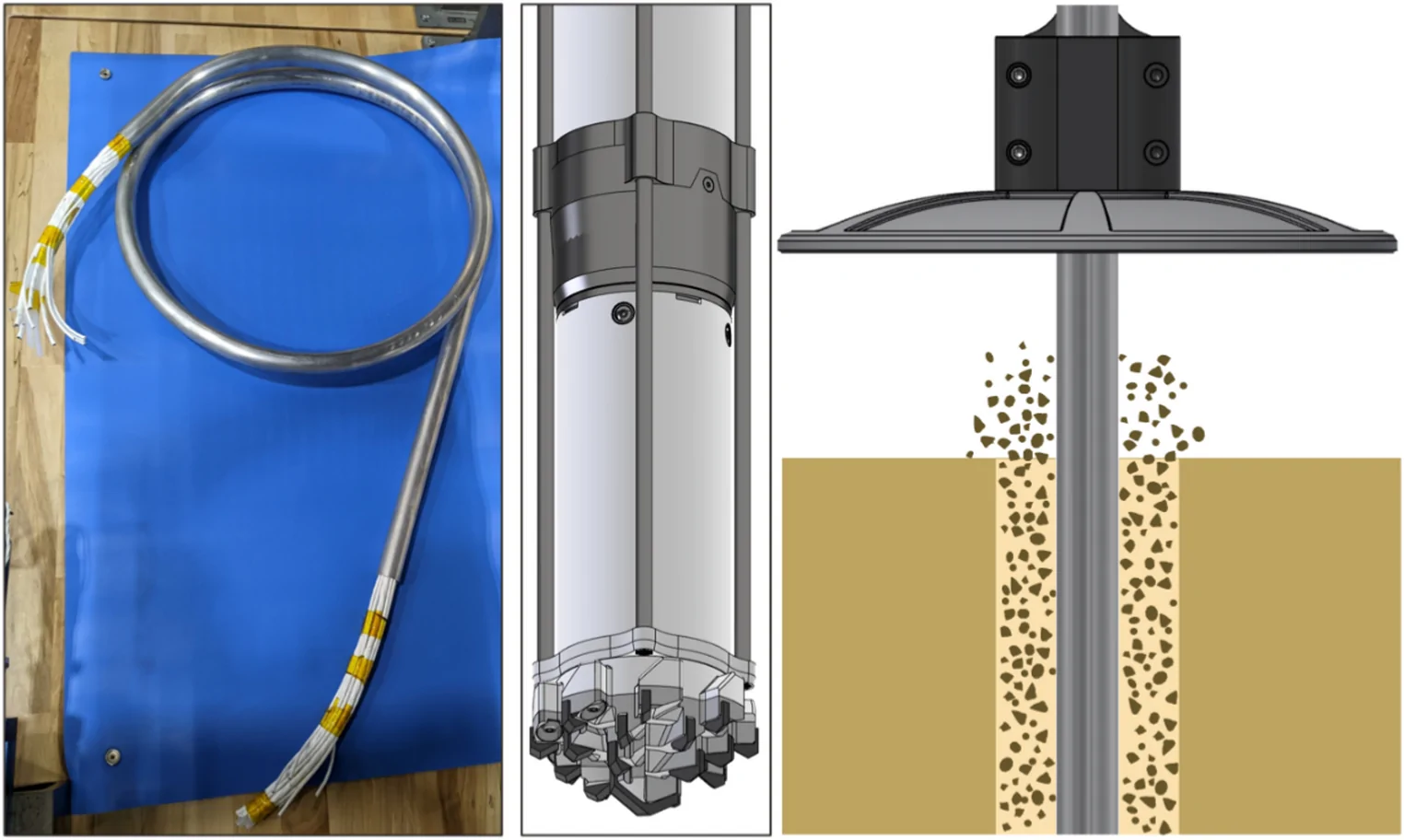
Images of RedWater subsystems relevant to the pneumatic drilling design. Left: Coiled tube hardware shown with the electrical and pneumatic and hydraulic lines fed through. Middle: Close up of RedWater drill bit, pneumatic lines, and pneumatic outlets for drill chip transport. Right: Diagram of chips transporting out of annular borehole, below a deflector plate that mounts to the coiled tubing

RedWater employs a direct circulation approach primarily for simplicity: the system requires only a single high-pressure gas supply line and avoids the complexity of downhole dual-wall pipe systems or bottom-hole assemblies with reverse flow paths. When drilling, gas is released from a high-pressure tank on the surface, where it flows through the coiled tubing and exits the system near the drill bit. The released gas entrains the drill chips and carries them up and out of the borehole (Fig. 4). Gas is either transported to Mars or captured and pressurized from the Martian atmosphere. Predicting the total mass of gas required for this operation is important to the RedWater mission architecture.

Pneumatic chip transport has been studied for terrestrial drilling applications, with early research establishing basic scaling between gas velocity, particle size, and annular geometry (Temple et al. 1996; Sharma and Chowdhry 1986). Approaches for characterizing flow regimes in vertical gas-solid systems utilize dimensionless regime maps based on the Reynolds number and modified Froude number or related dimensionless groups (Reh 1971; Grace 1986). These regime diagrams delineate the boundaries between flow states that describe vertical gas-solid systems as gas velocity increases: a packed bed of stationary particles, bubbling fluidization in which gas bubbles form and rise through the particle bed, slugging in which those voids grow to span the cross-section, and finally dilute-phase pneumatic transport in which particles are entrained and carried out of the system. Successful chip clearing requires operating in this last regime; the choking velocity marks the lower boundary below which flow collapses back into dense, recirculating slugging. Adewumi and Tian 1990; studied the important parameters for choking during chip transport and concluded that the coarsest particle size typically dominates required flow conditions. More recent analyses rely on Computational Fluid Dynamics (CFD) to model complex gas-solid flows for terrestrial deep drilling (Manjula et al. 2017).

Extraterrestrial applications of pneumatic chip clearing models are less studied. Zacny et al. 2005 produced an extensive test data set showing the effectiveness of intermittent gas bursts for clearing chips from depth of up to 25 cm. Tosi et al. (2024) employ a physics-based approach to approximate transport regimes for chip transport for a Martian deep drilling concept (Howe et al. 2022). Whereas Tosi et al. (2024) use dimensionless regime maps to classify particle transport behavior, the present work applies empirical pneumatic conveying correlations to directly compute minimum gas flow rates and system-level gas budgets for drilling on Mars, using a dimensionless regime map only to confirm that these results generalize across conditions.

## Materials and methods

### Pneumatic chip clearing model

The goal of the model is to estimate the required mass flow rate of gas required to adequately clear drill chips during the drilling operation. There are two regimes which drive the required gas input: (1) picking up and entraining chips at the bottom of the borehole, and (2) the pneumatic conveyance of the chips up and out of the borehole. The second regime is assumed to dominate the required gas, especially when the borehole is meters in depth.

To effectively convey particles up the borehole, the gas mass flow rate must be enough to prevent the choking condition, in which particle transport becomes inefficient, forms slugs or localized dense phases, and recirculates. The goal is to have just enough gas flow rate to keep dilute-phase flow in the borehole. While the choking phenomenon is not properly understood, it can be estimated with empirical equations. Several of these correlations exist and are summarized in Klinzing et al. 2010. This work uses the correlation of Punwani et al. (1976), which shows the lowest deviation between predicted and measured choking velocities among the correlations evaluated against experimental data from several sources (Klinzing et al. 2010; Table 5.2).

Correlations were developed for vertical pneumatic conveyance in round pipes for terrestrial environments. Since the open cross-sectional area in the borehole is an annulus, the equations must use the hydraulic diameter,$\:\:{D}_{H}={D}_{borehole}-{D}_{RW}$, and resultant area, $\:{A}_{H}$.

For shallow boreholes, the outer diameter of Redwater, $\:{D}_{RW}$, can be approximated as the diameter of the bottom hole assembly, as it takes up most of the borehole depth. For deeper boreholes, where the bottom hole assembly is fully submerged, the diameter of the coiled tubing will dominate, and should be used. The hydraulic area should not be confused with the actual area of the annulus, which is given as:

1 $$\:{A}_{A}=\frac{\pi\:}{4}({D}_{borehole}^{2}-{D}_{RW}^{2})$$

The choking gas velocity is given by the correlation of Punwani et al. (1976):

2 $$\:{v}_{c}=\sqrt{\frac{2g{D}_{H}\left(\epsilon_{c}^{-4.7}-1\right)}{\lambda\:}}+{w}_{fo\psi\:}$$

Voidage, *ε*, is defined as the ratio of gas-filled volume vs. particle volume. For dilute flow, this ratio is typically around 99%.

The choking voidage, $\epsilon_{c}$, is defined as the lowest voidage before the choking condition is initiated and is calculated from the Punwani et al. (1976) correlation.

3 $$\epsilon_{c}=1-\frac{j}{{\rho\:}_{p}\left({v}_{c}-{w}_{fo\psi\:}\right)}$$

The voidage and choking velocity equations are self-referencing, so they must be iteratively calculated until a value converges. The voidage depends on the mass flux of the solid drill chips being created while drilling, which must be calculated from the expected rate of penetration (ROP) of the drill.

4 $$\:j=\frac{{ROP*\:\frac{\pi\:}{4}{D}_{borehole}^{2}*\rho\:}_{p}}{{A}_{H}}$$

The choking velocity correlation also requires a friction factor, λ, which Punwani et al. (1976) empirically correlated to the gas density:

5 $$\:\lambda\:=.07{\rho\:}^{.77}$$

This empirical friction factor was developed as part of the choking correlation selected for its accuracy (see above). In this model, it is also used to estimate frictional pressure losses in the borehole (Eq. (14). Roughness-based friction models for open-hole drilling exist (e.g., Lyons et al. 2021) but require borehole surface characterization that is not available for this application. The Punwani correlation is retained for consistency with the choking formulation, and because the required gas mass flow rate is governed by the choking velocity rather than the friction loss term.

The free fall velocity of an individual particle must be known to calculate choking voidage. This free fall velocity depends on the particle Reynolds number and is given by the regime-based drag correlations in Table 1, compiled from Klinzing et al. (2010). The Stokes and Newton regime equations are classical results; the intermediate regime correlation follows Allen (1900), and the supercritical regime follows Clamen and Gauvin (1969). These equations require the dynamic viscosity of the gas, which can be obtained from reference correlations for common species such as nitrogen (Huber et al. 2024) and carbon dioxide (Laesecke and Muzny 2017).

**Table 1:** Free fall velocity equations for a range of flow regimes assessed using Reynolds number

Flow regime	Definition	Free fall velocity equation	
Stokes regime	* Re<2*	$\:{w}_{fo}=\frac{{d}^{2}\left({\rho\:}_{p}-\rho\:\right)g}{18\eta\:}$	(6)
Intermediate regime	* 0.5<Re<500*	$\:{w}_{fo}=\frac{.153{g}^{.71}{d}^{1.14}{\left({\rho\:}_{p}-\rho\:\right)}^{0.71}}{{\rho\:}^{.29}{\eta\:}^{.43}}$	(7)
Newton’s regime	$\:500<Re<2*{10}^{5}$	$\:{w}_{fo}=1.74{\left(\frac{d\left({\rho\:}_{p}-\rho\:\right)g}{\rho\:}\right)}^{.5}$	(8)
Supercritical regime	$\:Re>2*{10}^{5}$	$\:{w}_{fo}=3.65{\left(\frac{d\left({\rho\:}_{p}-\rho\:\right)g}{\rho\:}\right)}^{.5}$	(9)

The Reynolds number is calculated from the corrected free-fall velocity; because the velocity depends on the Reynolds number, the calculation is iterated until a value converges. The free fall velocity is then modified to account for the sphericity of the individual drill cutting using an empirical correlation from Klinzing et al. 2010.

10 $$\:{w}_{fo\psi\:}={w}_{fo}*0.843\mathrm{log}\frac{\psi\:}{.065}$$

Educated estimates must be made on the average equivalent diameter and sphericity of a drill chip and will depend on the parameters of the drilling system, the design of the drill bit, and the material being drilled. In short, these are estimates that must be made from testing. Choosing a larger particle diameter is recommended, as it is important to ensure there is enough flow to clear chips of all sizes. Drill cuttings are rarely round and can be estimated to have a sphericity of around 0.6 (Klinzing et al. 2010; Table 3.1).

The equations used to predict choking velocity have been compared to empirical observations; Klinzing et al. 2010 recommends applying a safety factor of 1.5 to obtain a minimum choking velocity design for a system. A drilling system that can provide enough gas to achieve this minimum gas velocity inside of the borehole will be able to efficiently clear the drilling chips. However, it is difficult to measure and design for a borehole gas velocity in an extraterrestrial environment. The more relevant parameter is the mass flow rate of gas. The mass flow rate of gas in the borehole across any given cross section is calculated as:

11 $$\:\dot{m}=\rho\:{A}_{A}{v}_{design}$$

Importantly, the density of the gas in the borehole is unknown. This density is required to calculate the Reynolds number, the particle free fall velocity, the friction factor, and the resultant mass flow rate, so it must be estimated for the model to be useful. The density can be estimated using the ideal gas law. This is an appropriate approximation here because the borehole gas pressure (on order of hundreds of pascals to a few kilopascals) is far below the critical pressure of CO_2_, so the compressibility factor remains near unity and real-gas effects are negligible. This estimate requires calculating the gas pressure in the borehole. An approach used for direct circulation drill modeling in Lyons et al. 2021 calculates the pressure in the bottom of the borehole by adding the tophole pressure (environmental pressure), the hydrostatic pressure from the gas in the borehole, and the pressure drop from friction in the borehole. Though the pressure is expected to change along the length of the borehole, the pressure at the bottom is used as a conservative estimate, since mass flow rate increase with density.

12 $$\:{P}_{borehole}={P}_{tophole}+{P}_{hydrostatic}+{P}_{friction}$$

The hydrostatic pressure is calculated by taking the average density of the gas and solid mixture in the borehole and multiplying by gravity and length of the borehole.

13 $$\:{P}_{hydrostatic}=[\rho\:{*\epsilon}_{c}+{{\rho\:}_{p}(1-\epsilon}_{c})]*gL\:\:$$

The pressure drop from friction in the borehole is calculated using the Darcy-Weisbach equation where the friction factor from Eq. (5) is used.

14 $$\:{P}_{friction}=\frac{\lambda\:L\rho\:{*v}_{design}^{2}}{{2D}_{H}}$$

Of course, the pressure is dependent on the density of the gas, so this must be solved iteratively. It is recommended to start the iteration at a gas density at the tophole pressure and temperature conditions. In this work, the iterative solution is considered converged when the relative change in gas density between successive iterations is less than 10^− 5^.

This minimum mass flow rate is a design parameter for the RedWater system that can be controlled and is calculated using the choked mass flow equation. This is not to be confused with the choking velocity, which is a phenomenon of dirty flow in vertical conveying. The choked mass flow equation calculates the mass flow rate of a gas through a choke point.

15 $$\:\dot{m}={C}_{D}{A}_{0}\sqrt{\gamma\:{\rho\:}_{0}{P}_{0}{\left(\frac{2}{\gamma\:+1}\right)}^{\frac{\gamma\:+1}{\gamma\:-1}}}$$

Therefore, the mass flow rate can be set by controlling the orifice size and geometry ($\:{C}_{D},{A}_{0}$), the upstream pressure ($\:{\rho\:}_{0},{P}_{0})$ and the gas type ($\:\gamma\:).$.

**Fig. 5:**
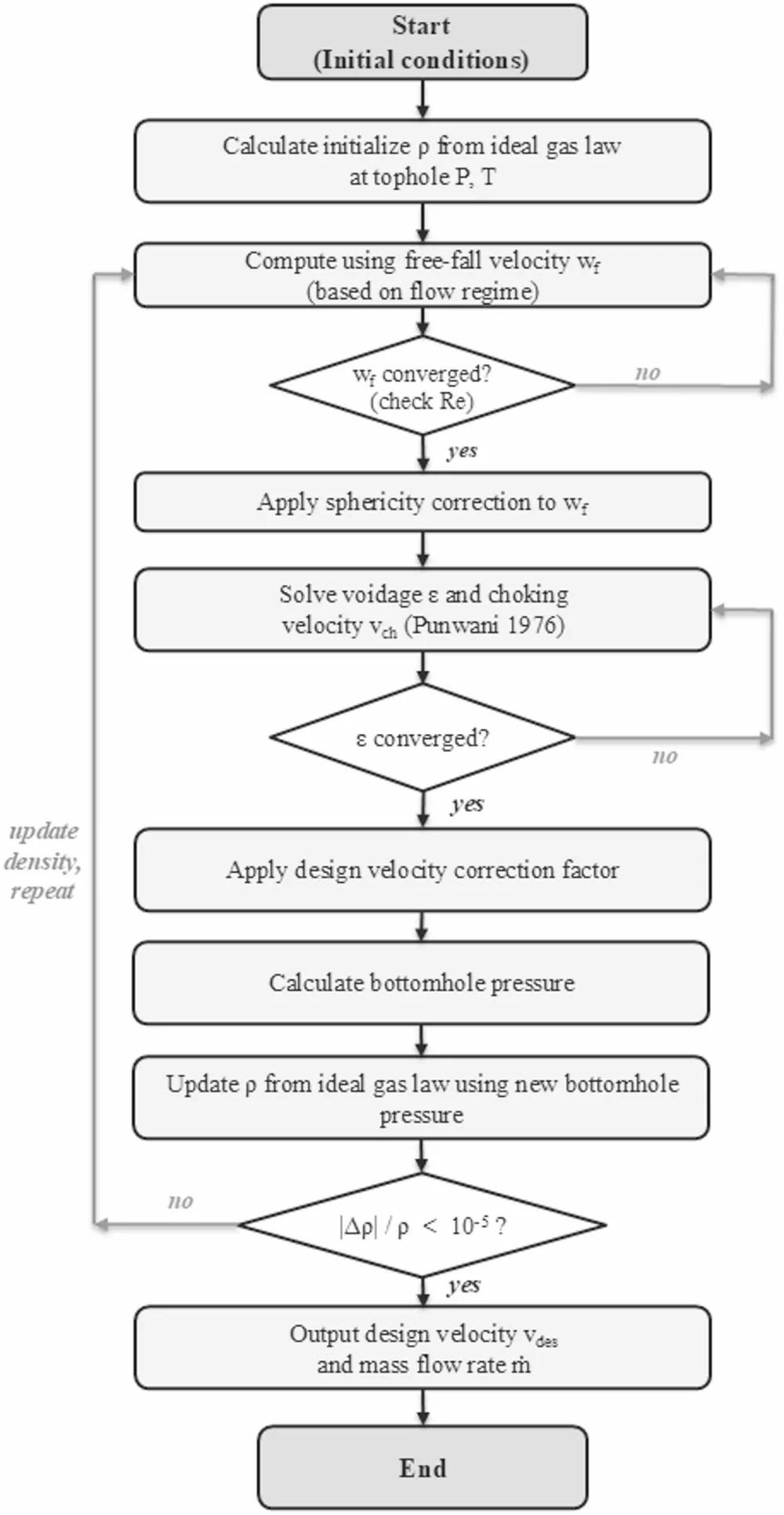
Flow chart for the pneumatic chip-clearing model, solved at each borehole depth. Three nested loops are evaluated: an inner loop that converges the regime-dependent free-fall velocity, a middle loop that converges the coupled voidage and choking velocity, and an outer loop that updates the gas density from the bottom-hole pressure. The converged solution yields the design velocity and the required gas mass flow rate

These coupled calculations are solved iteratively at each depth; the complete solution procedure, including the nested velocity, voidage, and density loops, is summarized in Fig. 5.

### Test setup and procedures

This model outputs are compared against several environments of varying geometries, chip properties, environmental conditions, and gas types. An initial bench-level test used silica sand to characterize the chip-clearing transition under controlled, repeatable particle conditions. The two subsequent system-level tests used actual water ice, including a thermal-vacuum test conducted with cryogenic ice under near-Martian pressure and CO_2_ gas, providing validation under conditions approaching those expected on Mars.

Because the pneumatic chip-clearing setup (Sect. Pneumatic chip clearing test setup) was the only test designed specifically for model validation, it is described here in the most detail. The two system-level tests (Sect. RedWater system-level freezer test setup and RedWater system-level TVAC test setup) were conducted to demonstrate end-to-end RedWater performance rather than to validate the pneumatic model; their full system design, instrumentation, and test procedures are reported in Mellerowicz et al. (2022) and Palmowski et al. (2024), and only the parameters relevant to chip clearing are summarized here.

#### Pneumatic chip clearing test setup

**Fig. 6:**
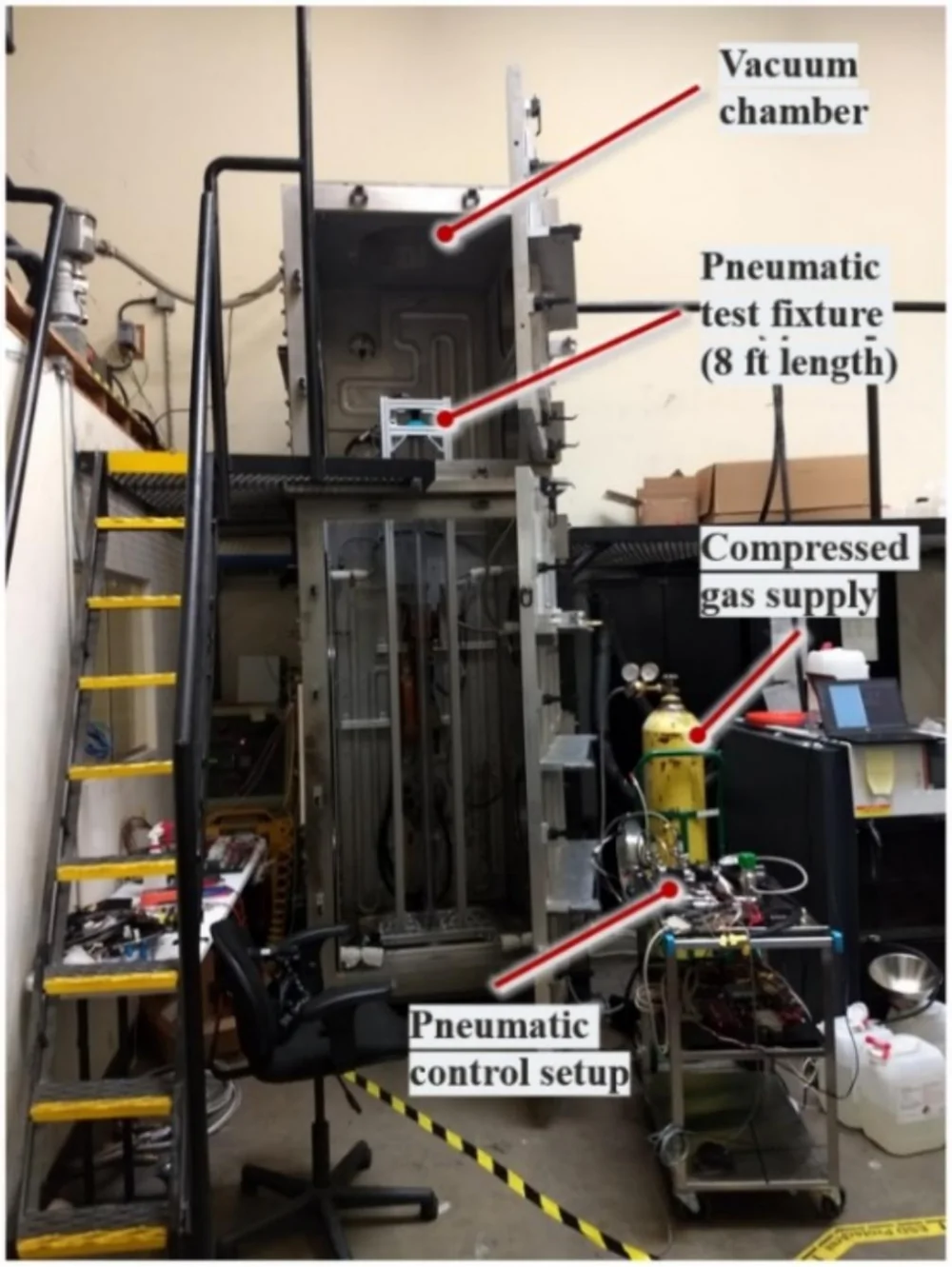
Setup to test pneumatic chip clearing in a Mars vacuum environment

A test setup was designed to replicate RedWater pneumatic chip clearing in a Martian vacuum environment (Fig. 6). The setup consisted of a vertical annulus 2.44 m long, formed by an inner tube of 38.1 mm diameter simulating the coiled tubing and an outer clear tube of 57.2 mm diameter simulating the borehole wall. These dimensions were based on early estimates of the RedWater borehole and coiled tubing diameters, before the prototype design was finalized. The outer tube was transparent to allow the chip clearing to be observed and recorded. Gas was delivered to the base of the annulus through 25 m of 6.35 mm (0.25 in) clear plastic tubing, representing the gas line running down the RedWater coiled tubing.

**Fig. 7:**
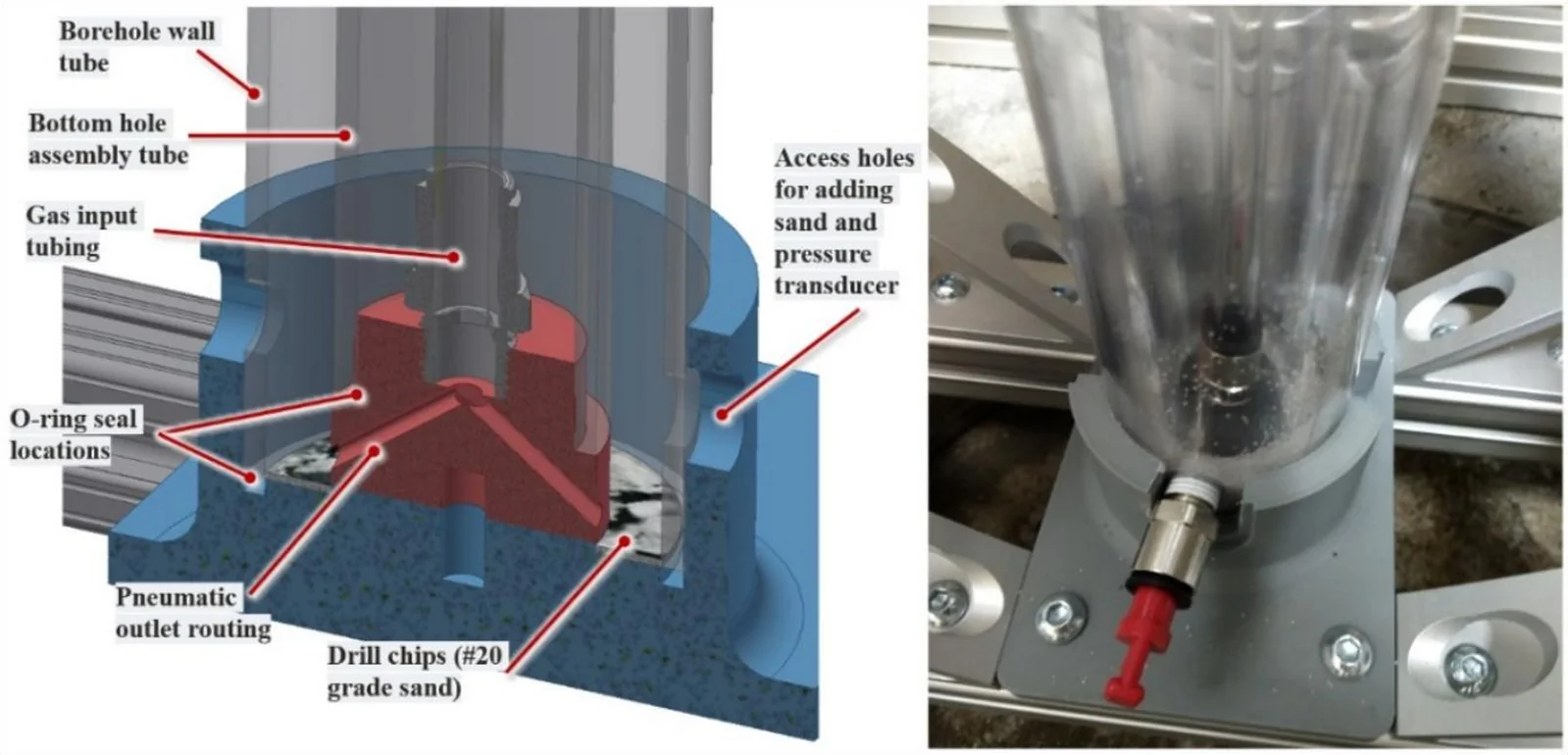
Left: Labeled CAD diagram of the bottom of the chip clearing test setup. Right: Image of the setup with simulant at the bottom of the annulus

Silica sand of known density and controlled particle size was placed at the bottom of the annulus to simulate drill chips accumulating in the borehole (Fig. 7). The sand was sieved to remove the finest particles. Silica sand was selected as a chip simulant for this initial test because its particle size and sphericity can be controlled and held consistent through sieving, unlike drill cuttings, allowing the chip-clearing transition to be characterized under repeatable conditions. Gas was supplied from a nitrogen tank through a fixed orifice at a regulated pressure. The mass flow rate was not measured directly but was calculated from the supply pressure and orifice geometry using the choked mass flow equation (Eq. (15). The vacuum chamber was held at 1.3 kPa by a controller and a vacuum pump. A camera recording at 30 frames per second viewed the bottom of the annulus to observe particle entrainment (Fig. 7).

Table 2 summarizes the parameters used for the test. The RedWater system architecture was still being defined during this phase, so a sample mass of 50 g was selected to represent the chips that would accumulate per pulse under a “bite” drilling approach under consideration at the time. The majority of tests during this campaign used a small plenum upstream of the annulus, which produced a time-varying mass flow rate unsuitable for determining a steady-state minimum. Three tests were therefore conducted at steady-state flow conditions, at 0.25, 0.4, and 1.0 g/s, to establish the minimum flow rate for efficient clearing. In the later system-level testing, the drilling operation was modified to flow gas continuously during drilling.

**Table 2:** Test conditions for the three chip clearing experiments

Parameter	Pneumatic chip clearing test	Freezer system test	TVAC system test
Environmental pressure	1.3 kPa (10 torr)	101 kPa (760 torr)	1.0 kPa (7.5 torr)
Environmental temperature	293 K	263 K	210 K
Drill chips	Sieved silica sand	Water ice	Cryogenic water ice
Chip density (kg/m^3^)	2000	916.8	916.8
Particle equivalent diameter (mm)	1.0	1.0 ^a^	0.5 ^a^
Sphericity	0.8	0.6	0.6
Rate of penetration (mm/s)	- ^b^	0.35–0.75 ^c^	0.5
Input gas	N_2_	N_2_	CO_2_
Input mass flow rate (g/s)	0.25, 0.4, 1.0 ^d^	16.7, 18.5 ^d^	0.55 ^e^

^a^Based on test observation and selection of larger chip sizes as conservative model input

^b^Not measured as a continuous rate; a 50 g sample was cleared in 10 s bursts (approx. 7.35 mm/s equivalent under the bite approach then considered)

^c^Range of ROP measured throughout the duration of the test

^d^Calculated from the regulated supply pressure and orifice geometry using the choked mass flow Eq. (15)

^e^Directly measured with an Alicat M-100SLPM flow sensor

#### RedWater system-level freezer test setup

**Fig. 8:**
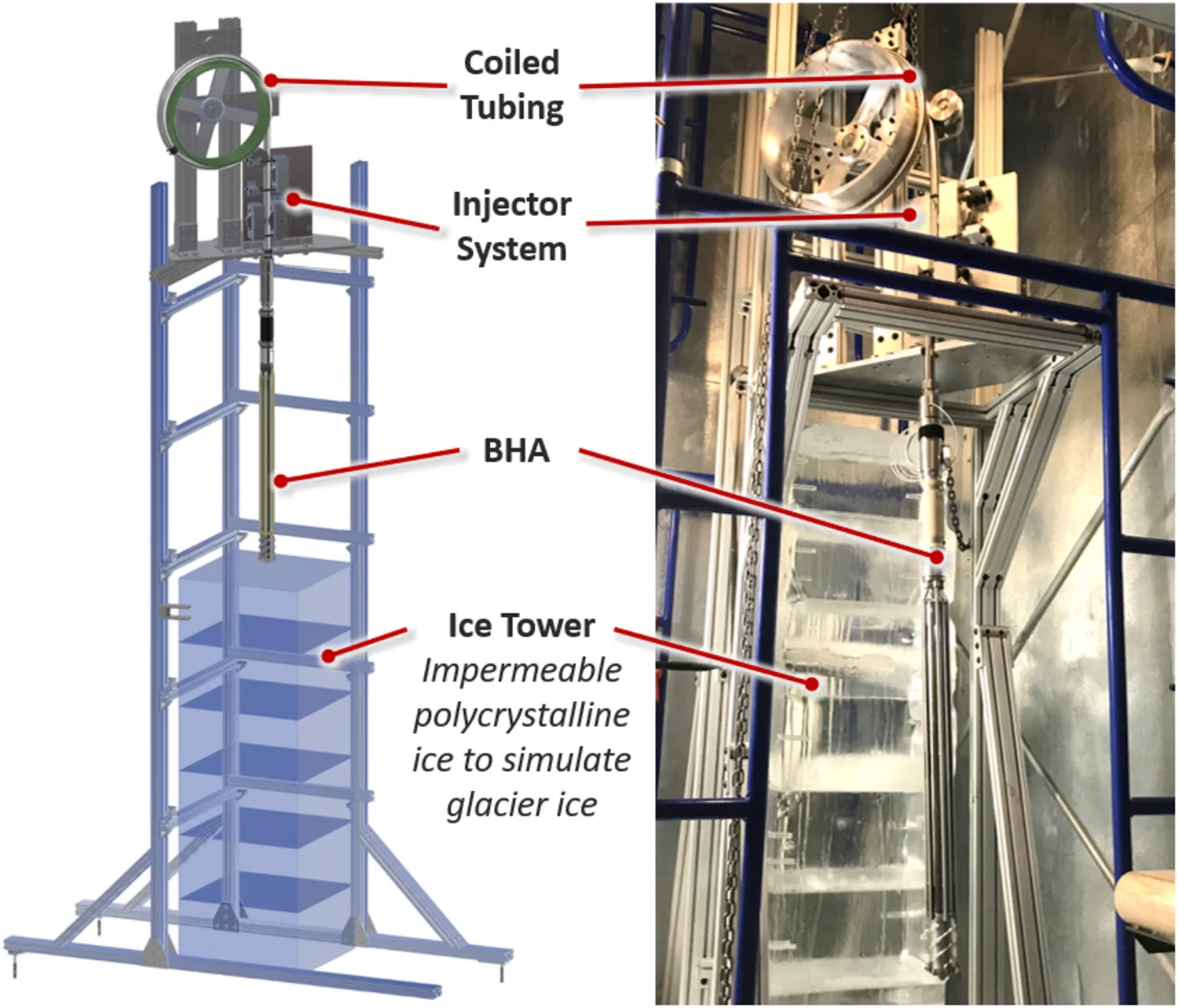
Left: CAD diagram of the RedWater system set up above an ice tower. Right: RedWater system before being mounted above the built ice tower inside the walk-in freezer

The priority for this test campaign was a successful end-to-end demonstration rather than pneumatic model validation. Nevertheless, the drilling phase provided a useful data point for the model. A build of the RedWater system was mounted over a crystalline tower of ice constructed inside a walk-in freezer (Fig. 8). The ice tower was built by stacking and fusing clear sculpture ice blocks, which provided a homogeneous, non-porous medium and a transparent wall through which chip transport in the borehole could be observed directly (Mellerowicz et al. 2022). Drilling used the RedWater Bottom Hole Assembly, with a 50 mm outer diameter operating in a borehole of approximately 72.5 mm diameter, producing a smaller annular gap than the dedicated chip-clearing setup. The initial supply pressure was set based on a preliminary pneumatic test in which the RedWater drill bit was mounted to a fixture and driven by a handheld rotary-percussive drill into crystalline ice, with the pneumatic supply pressure increased until cuttings began to clear from the bit. This test established a starting supply pressure of 550 kPa (80 psi); because it was performed by hand, weight-on-bit and rate of penetration were not recorded. The corresponding mass flow rate of 16.7 g/s was calculated from this supply pressure and the orifice geometry using the choked mass flow equation (Eq. 15). Nitrogen was used as the clearing gas, and drilling telemetry was logged from all system motors.

#### RedWater system-level TVAC test setup

**Fig. 9:**
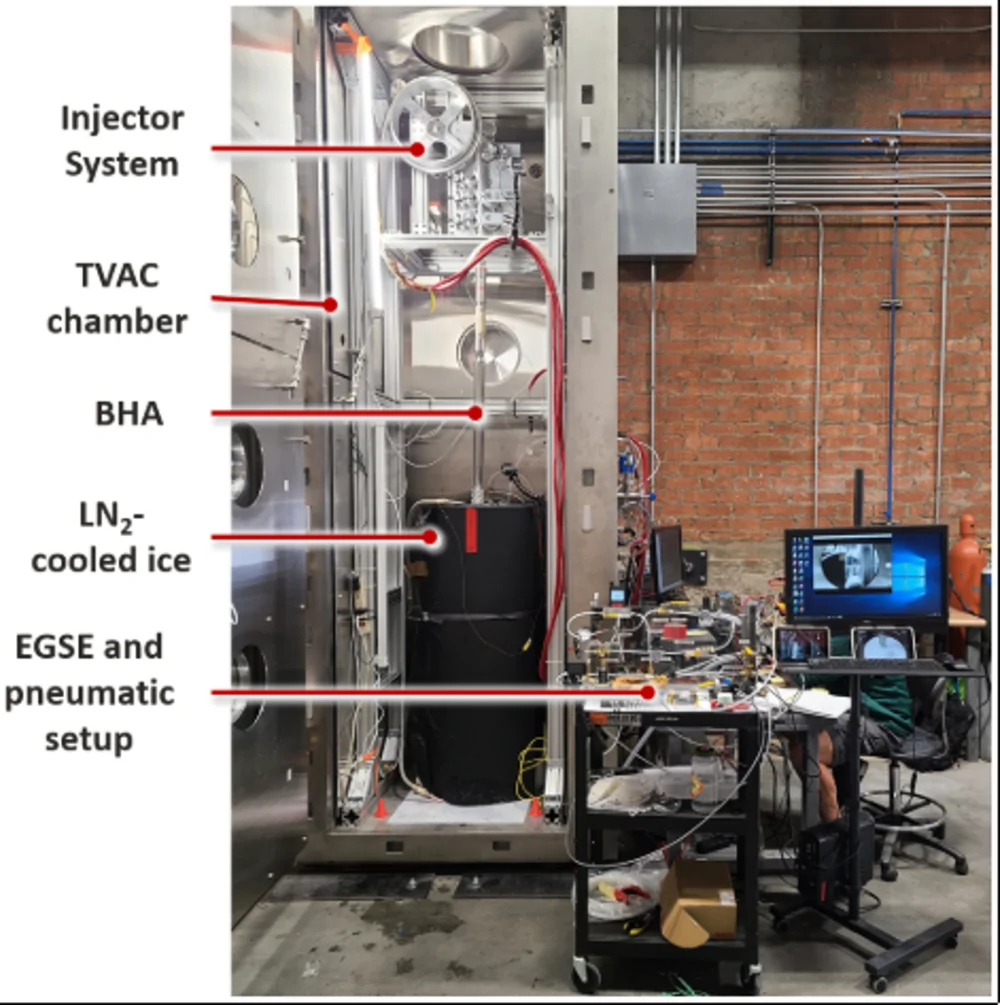
Image of the test setup for the RedWater system level TVAC test, before shutting the door to pull vacuum

The RedWater system was tested in a thermal vacuum (TVAC) chamber in cryogenic ice conditions (Fig. 9). This test replicated Martian ice conditions and demonstrated end-to-end performance resulting in liquid water delivered to a container outside of the vacuum chamber. Pressure was pumped down to 1.0 kPa (7.5 torr) and the ice was cooled to a temperature of ~ 210 K using liquid nitrogen. The mass flow rate was set to 0.55 g/s, directly measured with an Alicat M-100SLPM flow sensor. This value was selected before the test from a preliminary version of the model using rough estimates of ice temperature, ambient pressure, and rate of penetration, with additional margin applied. The model inputs were subsequently refined using the measured test conditions, geometry, and rate of penetration to produce the prediction reported in Sect. Comparison with model predictions.

As with the previous test, the goal of this test was not to validate the pneumatic chip clearing model, but to demonstrate full system performance. Table 2 shows the parameters from the test.

## Results

### Pneumatic chip clearing test results

**Fig. 10:**
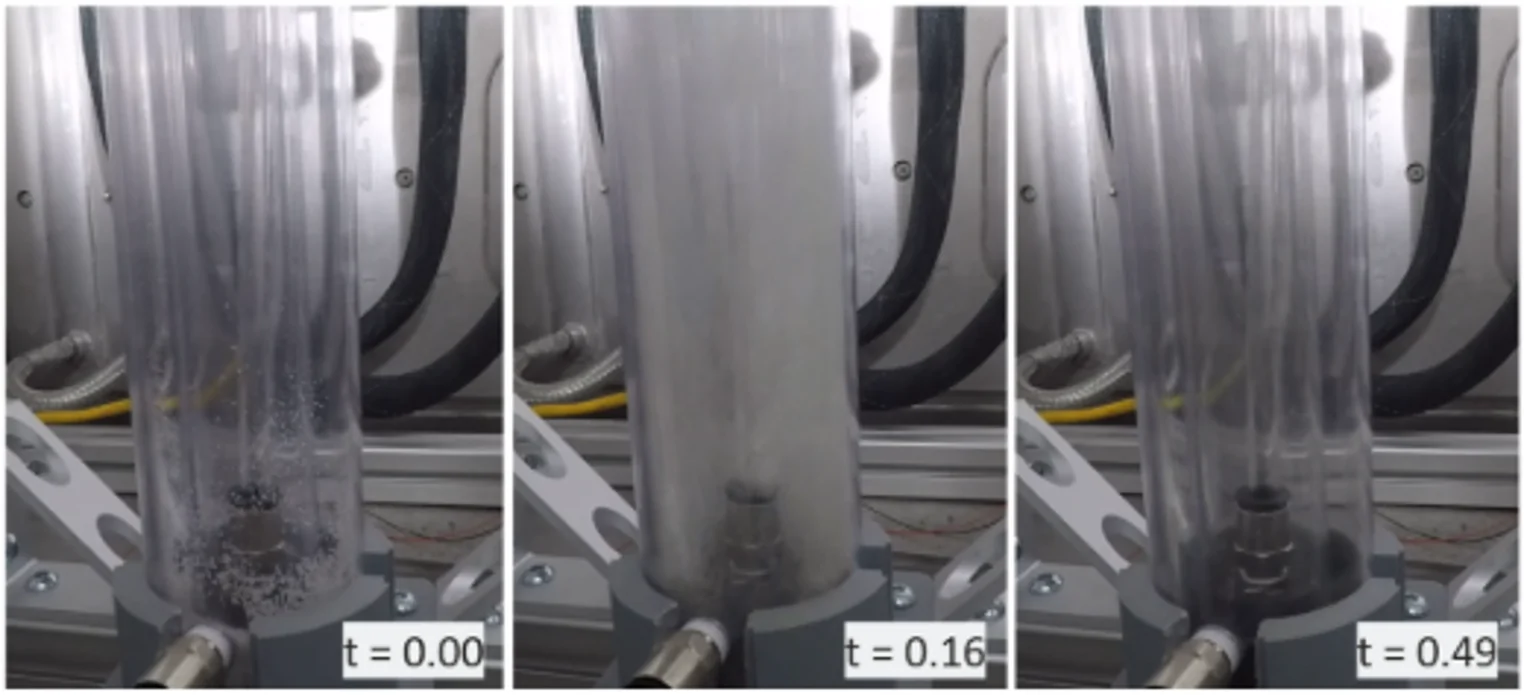
Video stills show an example of efficient chip clearing during a test in vacuum. The particles are entrained and entirely transported out of the frame in less than half a second

Video of the tests showed a clear difference between efficient and inefficient chip clearing. At 1.0 g/s input, the particles are all immediately entrained and moved out of the frame within 0.5 s (Fig. 10). At 0.25 g/s and 0.4 g/s, particles are observed to recirculate, indicating inefficient transport and a potential choking condition. After 10 s, the gas is turned off, and several of the particles are seen to remain in the annulus. Three tests were conducted to compare against each other, resulting in a wide range of a minimum mass flow rate for efficient chip clearing: between 0.4 g/s and 1.0 g/s.

### RedWater system level freezer test results

**Fig. 11:**
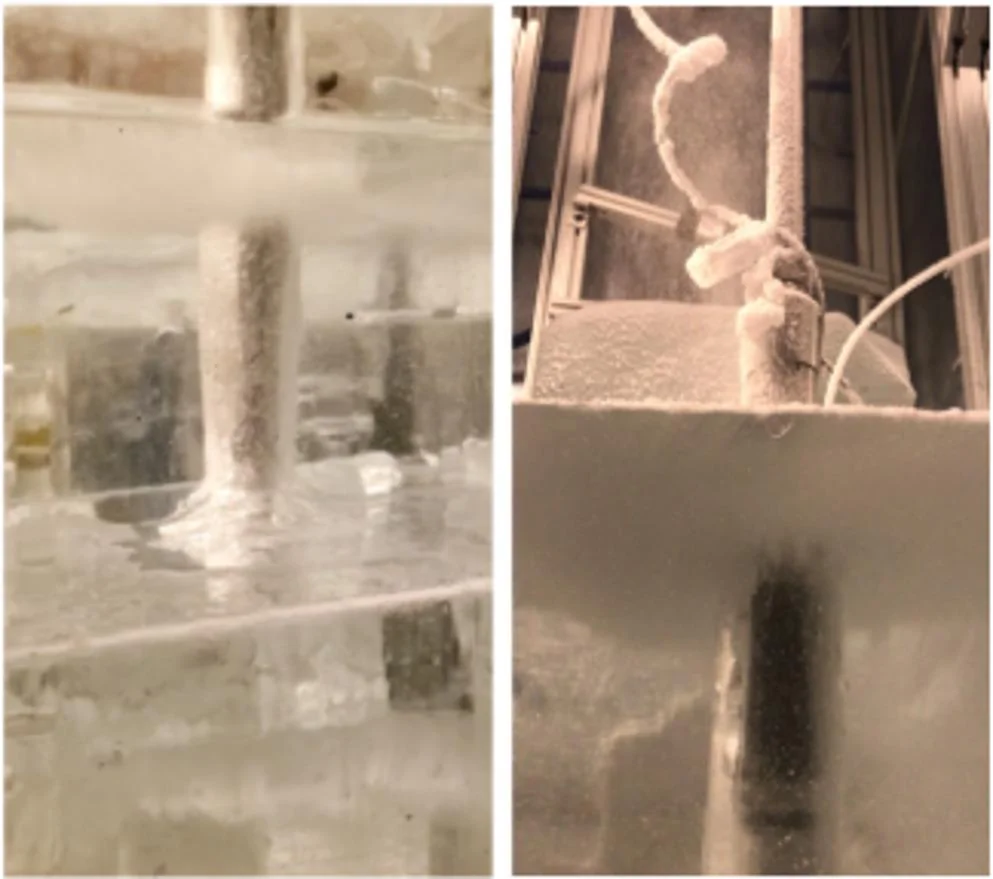
Left: View through the ice of the BHA drilling in the ice tower. Ice chips were observed to recirculate and inefficiently be transported up the annulus. Right: View of ice chips blowing out through the borehole after the flow rate was increased

The test successfully demonstrated RedWater end-to-end operations in ice at a temperature of −10 C, culminating in water delivered to an outside tank from a Rodwell created and pressurized inside the ice tower. The system drilled to a depth of 1.4 m at an average rate of penetration of 0.59 mm/s (Mellerowicz et al. 2022). The instantaneous rate of penetration varied between approximately 0.35 and 0.75 mm/s over the course of drilling; these bounds were used as model inputs to produce the predicted flow-rate range in Table 3. The test was conducted in an ambient pressure environment. Drilling telemetry data was collected on all the motors in the system. Throughout most of the test, the input flow rate was set from a high-pressure nitrogen tank, regulated down to 550 kPa (80 psi) and a mass flow rate of 16.7 g/s. Over time, some ice chip recirculation was visually observed in the borehole through the clear ice. The test was briefly paused due to a software error, at which point the team was able to increase the flow input to 690 kPa (100 psi) and 18.5 g/s. After this, chips were observed to blow out of the borehole with minimal recirculation (Fig. 11). The transition from recirculation to clearing between these two settings brackets the minimum flow rate used for comparison against the model.

**Table 3:** Comparison of model predictions to test results for minimum gas mass flow rates required to clear drill chips from the borehole

Test setup	Ambient pressure	Drill chips	Gas type	Model prediction (g/s)	Recirculation observed at (g/s)	Clearing observed at (g/s)
Pneumatic chip clearing test	1.3 kPa (10 torr)	Silica sand	N_2_	0.97	0.4	1.0
RedWater system-level freezer test	101 kPa (760 torr)	Ice	N_2_	16.4–16.9	16.7	18.5
RedWater system-level TVAC test	1.0 kPa (7.5 torr)	Cryo Ice	CO_2_	0.45	None observed	0.55

Ice chips created from the test were observed to range from very fine particles (< 0.5 mm) to larger ice chips (~ 1 mm). An estimate of 1 mm diameter was used for model comparison, though additional work is recommended to properly constrain particle size distribution. Table 2 shows the parameters from the test.

### RedWater system level TVAC test results

**Fig. 12:**
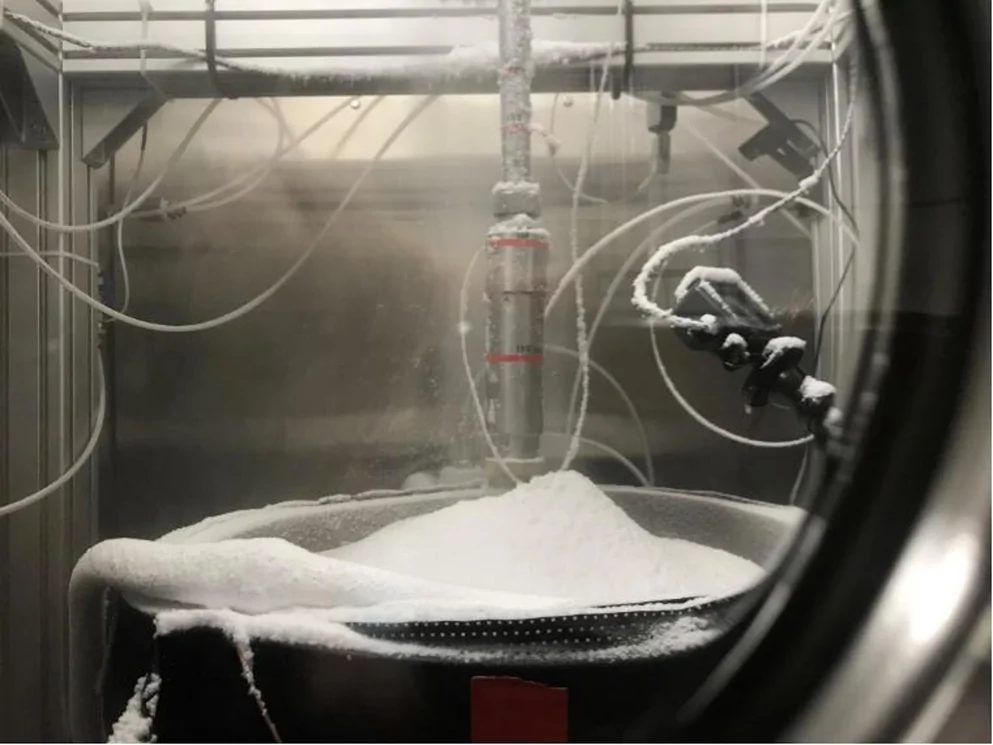
View of RedWater drilling through cryogenic ice inside the vacuum chamber. Ice chips are seen blowing out of the borehole and accumulating at the surface

During the TVAC test, a consistent CO₂ mass flow rate of 0.55 g/s was applied throughout drilling, and the ice chips were observed to blow out of the borehole without issue. Ice at cryogenic temperatures is more brittle (Arakawa and Maeno 1997), resulting in smaller ice chips generated from rotary percussive drilling (Fig. 12). Because the flow cleared the chips cleanly with no observed recirculation, this rate represents an upper bound on the minimum required flow rate rather than the threshold itself. Unlike the other two tests, in which the flow rate was calculated from the supply pressure and orifice geometry, the TVAC flow rate was directly measured, providing the most tightly constrained input of the three comparisons. As with the freezer test, the objective was full-system demonstration rather than model validation; nevertheless, it provides a useful data point at near-Martian pressure with CO_2_.

### Comparison with model predictions

The measured conditions from each test - geometry, gas type, ambient pressure, chip properties, and rate of penetration - were used as inputs to the model to predict the minimum gas mass flow rate required for chip clearing. Table 3 compares each prediction against the flow rates observed to recirculate (insufficient clearing) and to clear chips. Where both were recorded, the true minimum clearing flow rate lies between them; for the TVAC test, only a single clearing flow rate was applied, providing an upper bound. The interpretation of these comparisons is discussed in Sect. Interpretation of model–test comparison.

## Discussion

### Interpretation of model-test comparison

The model reproduces the required clearing flow rate across a range of flow rates, despite large differences in input parameters. For the two Mars-relevant low-pressure tests, the prediction is consistent with the observations: the sand chip clearing test prediction (0.97 g/s) falls within the observed transition between recirculation and clearing, and the TVAC prediction (0.45 g/s) lies just below the single rate confirmed to clear chips (0.55 g/s). For the freezer test, the prediction (16.4–16.9 g/s) sits just below the 16.7 g/s rate at which recirculation was still observed, placing the true minimum only slightly above the prediction. The confirmed-clearing rate of 18.5 g/s does not constrain the minimum closely, because no flow rates were tested between 16.7 and 18.5 g/s, and the true threshold could lie anywhere in that interval, including near the model prediction.

Taken together, these comparisons support the modeling approach: terrestrial dilute-phase pneumatic conveying correlations, applied with Mars-relevant inputs, reproduce the observed clearing flow rates across different gases, ambient pressures, and chip materials. The largest difference in required gas mass flow rate among the tests (more than 30x between the 18.5 g/s for the freezer test at Earth-ambient pressure and the 0.55 g/s for the TVAC test at near-Martian pressure) is driven primarily by ambient pressure through its effect on required gas density to clear drill chips. The model reproduces both ends of this range from a single framework. This supports pneumatic drilling as a viable approach for the Martian environment.

This reduction in required gas can be quantified directly from the total gas consumed during drilling, providing a system-level measure of performance alongside the flow-rate threshold. Expressed as the mass of cuttings cleared per unit mass of gas used, the freezer test measured 0.16 kg/kg at Earth-ambient pressure, whereas the TVAC test measured 3.6 kg/kg at near-Martian pressure (Mellerowicz et al. 2022); a difference of more than 20x. This efficiency gain reflects the same pressure-driven reduction in required flow rate noted above, now expressed as gas consumed per unit mass of cuttings cleared. The measured TVAC value also falls at the lower end of the 3.0–23.8 kg/kg range the model predicts for full-scale Martian drilling (Table 4), providing an order-of-magnitude consistency check, although that range assumes a basaltic overburden rather than ice.

### Practical application to RedWater

Table 4 shows model predictions for a notional RedWater system on Mars under several assumptions for average chip properties. The diameter of the coiled tubing is used for this estimate as it will dominate at deeper depths.

**Table 4:** Assumed input parameters and predicted gas requirements for a notional RedWater pneumatic drilling system on Mars. Outputs assume drilling through basaltic rock only and normalized to 1 meter depth

Parameter	Property or predicted range	Note
Overburden composition	Mars basaltic overburden	Assuming only drilling through rock (conservative)
Rock density	2900 kg/m^3^	Estimate based on study from (Lakshmi and Kumar 2020)
Average chip equivalent diameter	1 mm	Estimate from testing
Average chip sphericity	0.6	Estimate from testing
Gas type	CO2	Compressed from Martian atmosphere
Atmospheric pressure	600 Pa	Based on Mars atmospheric density measured by the Phoenix Lander (Martínez et al. 2017)
Rate of penetration	0.35–3 mm/s	Estimated range from testing
Required gas flow estimate from model	1.38–1.51 g/s	–
Total required gas per meter drilled	3.95–0.50 kg/m	–
Mass excavated per mass of gas supplied	3.0–23.8 kg/kg	Assumes a column of rock at 2900 kg/m^3^

**Fig. 13:**
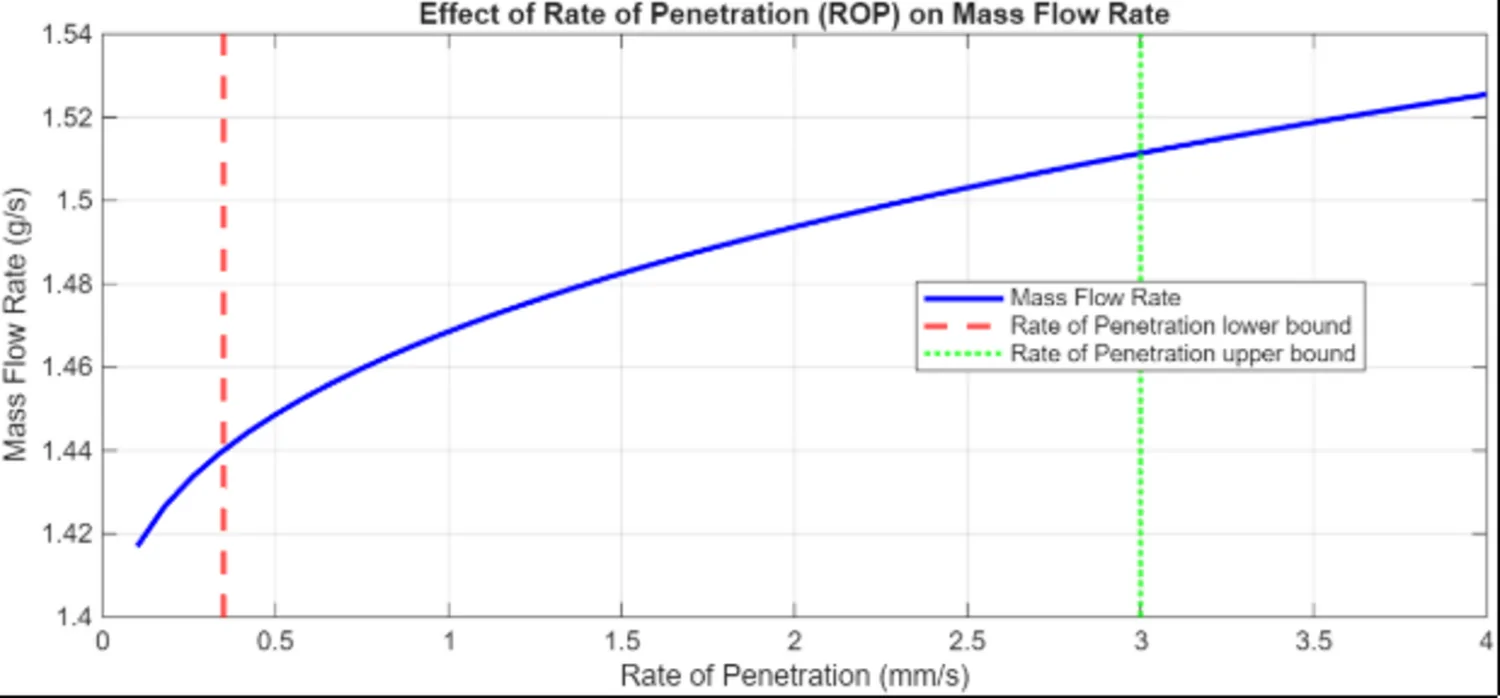
Sensitivity analysis showing the effects of drill speed, or rate of penetration (ROP), on the minimum mass flow rate to clear chips for the notional RedWater pneumatic drilling system on Mars. The lower and upper bounds of drilling speeds are based on test results

Interestingly, the required flow rate does not strongly depend on rate of penetration. The voidage remains high (> 0.99) even at a faster drill speed that produces more cuttings. Figure 13 shows how the required mass flow rate changes with rate of penetration in a range that may be expected for the drilling system.

While the mass flow rate is not significantly driven by the rate of penetration, the total required gas per meter drilled is. This highlights the importance of a rapid drilling system as it saves on total mass of gas. Alternatively, a pulsed chip clearing architecture may be more efficient than one that is always on. Allowing chips to build up in the borehole will not significantly affect the mass flow rate, meaning more material can be cleared per mass of gas. The length of the pulse will depend on the depth of the borehole, as it must be long enough to transport the entrained chips to the surface.

**Fig. 14:**
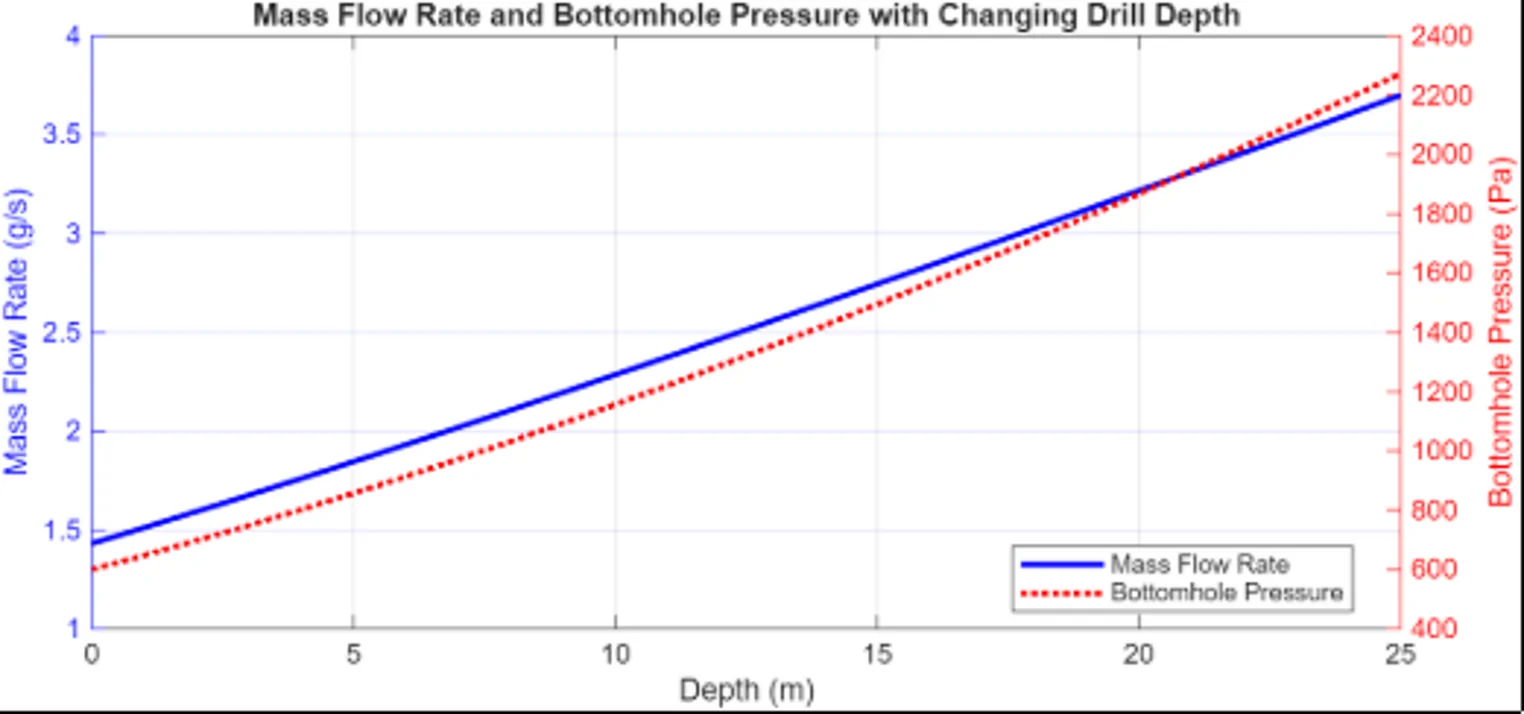
Changing bottomhole pressure and mass flow rate with respect to borehole depth notional RedWater pneumatic drilling system on Mars

Figure 14 shows the effect of depth on bottomhole pressure and mass flow rate. As RedWater drills deeper, both hydrostatic and frictional pressure drop increase, resulting in a higher required mass flow rate to clear chips.

To test whether these results generalize beyond the specific conditions tested, the operating points were recast in dimensionless form. All four cases sit in the intermediate particle-drag regime defined by the particles Reynolds number (2 < Re_p_ < 500), with sand test at Re_p_ = 24, the freezer test at Re_p_ = 425, the TVAC test at Re_p_ = 4, and the Mars projection at Re_p_ = 28. Each also sits at a Froude number well above one (13 to 85), where gas inertia dominates particle settling.

**Fig. 15:**
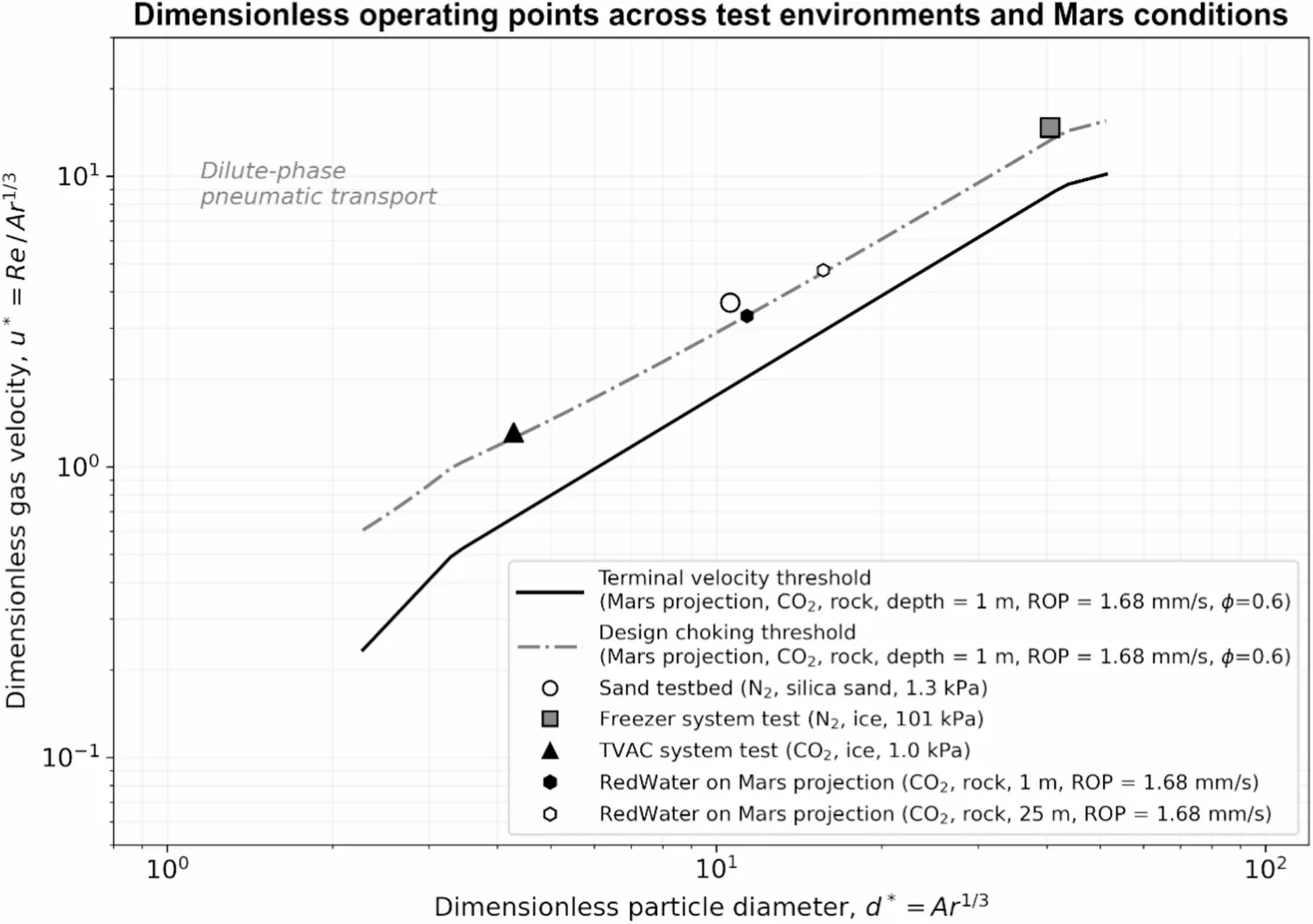
Dimensionless operating points across test environments and Mars conditions, on the generalized flow regime diagram (Grace 1986). Axes are the dimensionless particle diameter d* = Ar^1/3^ and dimensionless gas velocity u* = Re/Ar^1/3^. Each marker is a design operating point computed from the conditions in Table 2 (tests) and Table 4 (Mars projection). Both threshold lines are computed from Mars projection values at an assumed ROP of 1.68 mm/s; the average of the ROPs observed during testing

Figure 15 places these points on the generalized flow regime diagram of Grace (1986), which maps gas-solid systems onto regions of distinct flow behavior (packed bed, fluidization, and dilute-phase transport) using two dimensionless coordinates. Both boundaries, the single-particle terminal velocity and the design choking threshold, are computed from the present model rather than from external correlations. The knees in these boundaries occur where the particle Reynolds number crosses between the drag regimes from Table 1. Despite spanning two gases, two gravities, and three geometries, every point falls in the dilute-phase transport region, above the terminal-velocity boundary. The sand test and the Mars projection nearly coincide (d* ≈ 11), indicating the sand test a useful Mars analog, while the TVAC test, run in CO₂ near Martian pressure, sits closest to the Mars choking threshold.

The model’s applicability is therefore set by the flow regime rather than the specific gas, gravity, or geomtery. This also holds with depth: as RedWater drills deeper the operating point moves to higher Re_p_ but stays in the same intermediate-drag, dilute-phase regime, so the model remains valid over the full drilling interval.

Given this confidence in the model, the required gas for RedWater pneumatic drilling on Mars is achievable. The gas supply can either be brought as part of the mission mass or compressed on the Martian surface. The Mars Oxygen ISRU Experiment (MOXIE) has demonstrated the compression of atmospheric Martian gas through the use of a scroll pump (Hoffman et al. 2023). Drilling through a meter of rocky overburden requires less than a kilogram of gas for an optimized system. Orbital datasets show that there are many locations where subsurface ice deposits are within less than 5 m of overburden (Putzig et al. 2023; Morgan et al. 2025). This gas requirement should be accounted for in architectural studies either with landed mass or power budgets for compressing gas.

### Limitations and future work

While the model performs well under the tested conditions, several limitations should be considered. Chip clearing is fundamentally governed by the balance between the aerodynamic drag the gas exerts on a particle and the particle’s weight, with the choking condition marking where that balance fails. The model captures the first-order parameters that set this balance (gas parameters, particle size, density, and gravity). Its accuracy, however, is bound by the empirical nature of the choking correlations, whose fractional exponents reflect fitted corrections rather than first-principles physics, and by the uncertainty in estimated particle size and sphericity. These correlations were derived from terrestrial, Earth-ambient testing, and their extrapolation to Martian conditions should be validated more directly.

The experimental tests were not specifically designed for model validation, as they were conducted independently to assess RedWater system performance. The test data represent visual observations of successful chip clearing rather than precise measurements of minimum flow thresholds, contributing to the wide ranges observed in some results. With only three validation points available, statistical confidence intervals cannot be established.

**Fig. 16:**
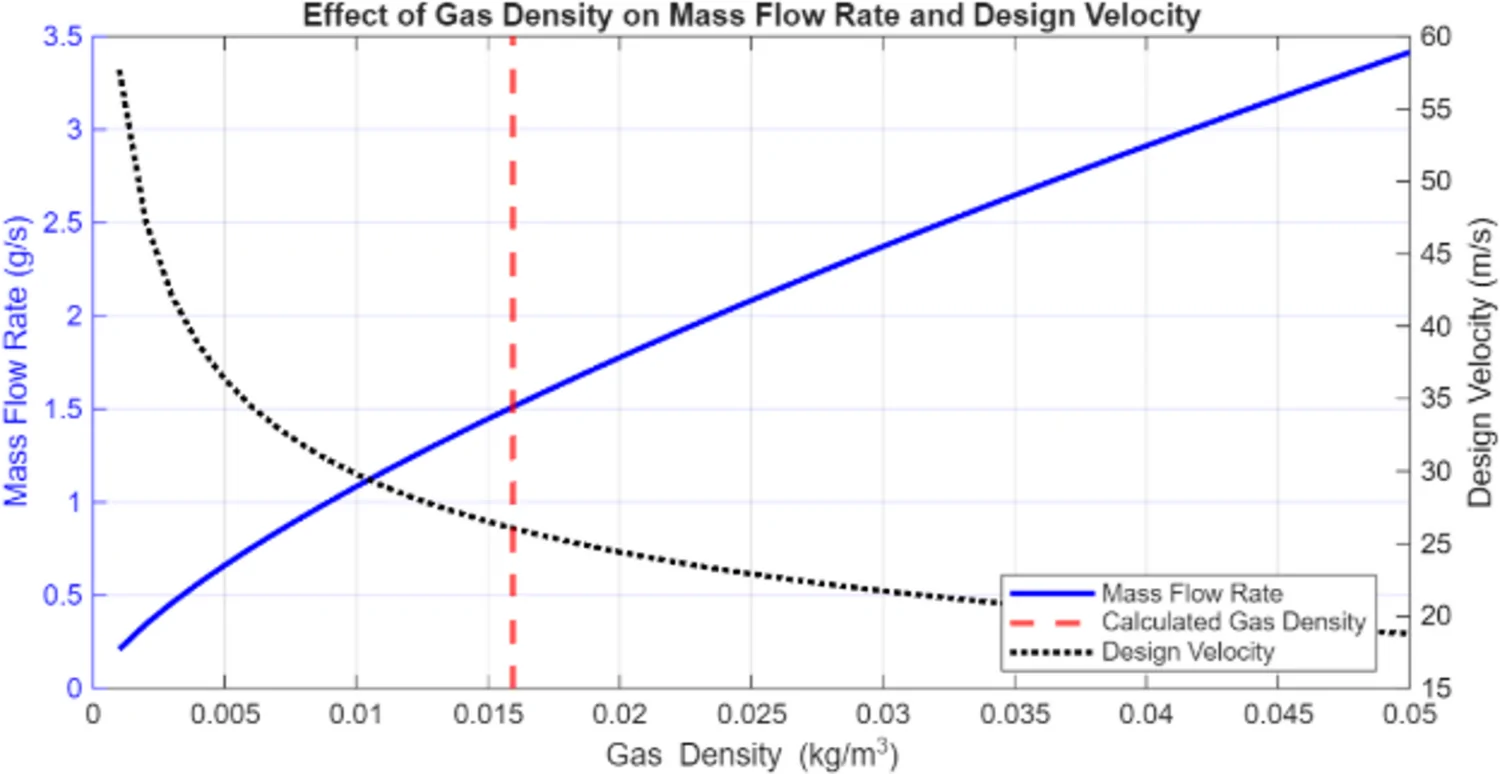
Sensitivity analysis showing the importance of gas density on minimum mass flow rate and velocity for chip clearing for the notional RedWater pneumatic drilling system on Mars

The model also requires simplifying assumptions that may not fully capture the dynamics of the drilling system. For example, both borehole and bottom hole assembly (BHA) geometries are idealized as perfect circles, and drill chips are assumed to be uniform in size and sphericity. The model also treats cuttings as discrete particles in dilute-phase transport and does not capture particle cohesion, which may occur with ice cuttings and could affect pickup at the bottom of the borehole. No transport failure attributable to ice cohesion was observed during the system-level ice tests, but this effect warrants dedicated investigation. The model is also sensitive to the estimated gas density: Fig. 16 shows the effect of gas density in the borehole on the designed gas velocity and the required mass flow rate. While the gas density is calculated in the model, it depends on several inputs that must be estimated, such as gas temperature and frictional pressure losses in the borehole.

One key model input remains unconstrained by these tests: gravity. All three experiments were conducted at Earth gravity, whereas the model predicts that Mars’s lower gravity (0.38 g) would further reduce the required gas mass flow rate by lowering the effective weight of the cuttings.

A further limitation is that the model assumes all supplied gas contributes to chip transport in the annulus. In practice, the borehole wall is rock and ice that may be porous or fractured, and some gas can be lost into the surrounding formation rather than returning up the annulus. Such losses would reduce the effective gas flow available for chip clearing and would raise the supply rate required to maintain transport, particularly in fractured overburden or porous ice.

The present model also terminates at the borehole exit and therefore does not represent a complete surface cuttings management system. In practice, cuttings discharged by the gas flow would need to be diverted or further transferred away from the borehole, as material accumulation near the borehole mouth could obstruct the flow path and reduce circulation. This is identified as a system-level design consideration beyond the scope of the present model.

Several aspects fall outside the scope of the present study and are identified here to guide future work. First, the model has been compared only against bench and system-level tests at sub-meter to meter depths; full-scale validation at the borehole lengths envisioned for RedWater (up to 25 m) is needed to confirm that the predicted pressure losses and transport behavior hold as the annulus lengthens. Second, the experiments used here were not designed for model validation and provide only bracketed or one-sided bounds on the clearing threshold; dedicated tests that finely resolve the transition between recirculation and clearing would substantially strengthen the comparison. Third, computational fluid dynamics (CFD) simulation of the gas-solid flow in the annulus would provide an independent check on the empirical correlations and capture effects the present model idealizes, such as non-uniform particle size, irregular borehole geometry, and the entraining flow near the bit. Fourth, because gravity cannot be reduced in a ground-based chamber, validating the predicted effect of Mars’s lower gravity would require reduced-gravity testing, such as parabolic-flight pneumatic transport experiments (Zacny et al. 2010). Finally, all testing to date has used prepared, homogeneous ice and sand; testing with realistic regolith, ice with dust content, mixed particle sizes, and porous or fractured formations is recommended to better assess performance under the conditions expected on Mars.

Nevertheless, the agreement across different test configurations, scales, and operating conditions suggests the model captures the governing physics and provides sufficient accuracy for system-level gas requirement estimates, which is appropriate given the current stage of technology development.

## Conclusion

This study develops and applies a pneumatic chip clearing model for the RedWater ice mining system, targeting deep drilling operations on Mars. Chip clearing is a necessary requirement for any drilling system and must therefore be accounted for in the development of any planetary deep drill. By leveraging empirical correlations for vertical pneumatic conveying to Martian conditions, the model provides gas flow estimates in reasonable agreement with experimental results, supporting preliminary system design for the RedWater drill and any other planetary pneumatic drilling systems. The model was tested against three experimental setups relevant to expected Martian conditions, each with a unique set of inputs. Due to the reliance on assumptions on gas and particle properties, and the dependence on Earth-based empirical equations, it is recommended more targeted model validation testing is conducted in future work. Despite these limitations, the model is sufficient for informing system-level requirements at this stage.

## Data Availability

The datasets generated and analyzed during the current study are available from the corresponding author on reasonable request.
